# Cohort profile: Understanding the influence of early life environments and health and social service system contacts over time and across generations through the Western Australian Aboriginal Child Health Survey (WAACHS) Linked Data Study

**DOI:** 10.1136/bmjopen-2024-087522

**Published:** 2024-10-02

**Authors:** Francis Mitrou, Helen Milroy, Juli Coffin, Sharynne L Hamilton, Christopher G Brennan-Jones, Stefanie Schurer, Elizabeth A Davis, Peter Richmond, Hayley M Passmore, Glenn Pearson, Alex Brown, Melissa O'Donnell, Asha C Bowen, Peter Azzopardi, Katherine M Conigrave, Jenny Downs, Matthew M Cooper, Kathryn A Ramsey, Anna Ferrante, Sarah E Johnson, Leah Cave, Philip Vlaskovsky, Katrina D Hopkins, Heather A D'Antoine, Ted Wilkes, Stephen R Zubrick

**Affiliations:** 1 Population Health, The Kids Research Institute Australia, Perth, WA, Australia; 2 Centre for Child Health Research, The University of Western Australia, Perth, WA, Australia; 3 Medical School, Psychiatry, The University of Western Australia, Perth, WA, Australia; 4 Mental Health and Youth Program, The Kids Research Institute Australia, Perth, WA, Australia; 5 Ngangk Yira Institute for Change, Yawardani Jan-ga Research Centre, Murdoch University, Broome, WA, Australia; 6 University of Canberra, Canberra, ACT, Australia; 7 Indigenous Health, The Kids Research Institute Australia, Perth, WA, Australia; 8 Ear Health Team, The Kids Research Institute Australia, Perth, WA, Australia; 9 Faculty of Health Sciences, Curtin University, Perth, WA, Australia; 10 School of Economics, The University of Sydney, Sydney, NSW, Australia; 11 Diabetes and Obesity Research Team, The Kids Research Institute Australia, Perth, WA, Australia; 12 Medical School, Paediatrics, The University of Western Australia, Perth, WA, Australia; 13 Wesfarmers Centre for Vaccines and Infectious Diseases, Telethon Kids Institute, Perth, WA, Australia; 14 Law School, The University of Western Australia, Nedlands, WA, Australia; 15 Indigenous Genomics, Australian National University, Canberra, ACT, Australia; 16 Australian Centre for Child Protection, University of South Australia, Adelaide, SA, Australia; 17 Centre for Adolescent Health, Murdoch Children's Research Institute, Department of Paediatrics, The University of Melbourne, Melbourne, Victoria, Australia; 18 Population Health, The Kids Research Institute Australia, Adelaide, SA, Australia; 19 Faculty of Medicine and Health, Central Clinical School, NHMRC Centre of Research Excellence in Indigenous Health and Alcohol, The University of Sydney, Sydney, NSW, Australia; 20 Development and Disability, The Kids Research Institute Australia, Perth, WA, Australia; 21 Biometrics, The Kids Research Institute Australia, Perth, WA, Australia; 22 Children's Lung Health, The Kids Research Institute Australia, Perth, WA, Australia; 23 Faculty of Health Sciences, School of Population Health, Curtin University, Perth, WA, Australia; 24 Centre for Applied Statistics, The University of Western Australia, Perth, WA, Australia; 25 Menzies School of Health Research, Darwin, NT, Australia; 26 School of Indigenous Studies, The University of Western Australia, Perth, WA, Australia; 27 Australian Institute of Health and Welfare, Canberra, ACT, Australia

**Keywords:** EPIDEMIOLOGY, PUBLIC HEALTH, Health Equity, Child, Family

## Abstract

**Abstract:**

**Purpose:**

Despite the volume of accumulating knowledge from prospective Aboriginal cohort studies, longitudinal data describing developmental trajectories in health and well-being is limited. The linkage of child and carer cohorts from a historical cross-sectional survey with longitudinal health-service and social-service administrative data has created a unique and powerful data resource that underpins the Western Australian Aboriginal Child Health Survey (WAACHS) linked data study. This study aims to provide evidence-based information to Aboriginal communities across Western Australia, governments and non-government agencies on the heterogeneous life trajectories of Aboriginal children and families.

**Participants:**

This study comprises data from a historical cross-sectional household study of 5289 Aboriginal children from the WAACHS (2000–2002) alongside their primary (N=2113) and other (N=1040) carers, and other householders. WAACHS data were linked with Western Australia (WA) government administrative datasets up to 2020 including health, education, child protection, police and justice system contacts. The study also includes two non-Aboriginal cohorts from WA, linked with the same administrative data sources allowing comparisons of outcomes across cohorts in addition to between-group comparisons within the Aboriginal population.

**Findings to date:**

Linked data coverage rates are presented for all WAACHS participants. Child health outcomes for the WAACHS children (Cohort 1) are described from birth into adulthood along with other outcomes including child protection and juvenile justice involvement.

**Future plans:**

Analysis of data from both the child and carer cohorts will seek to understand the contribution of individual, family (intergenerational) and community-level influences on Aboriginal children’s developmental and health pathways, identify key developmental transitions or turning points where interventions may be most effective in improving outcomes, and compare service pathways for Aboriginal and non-Aboriginal children. All research is guided by Aboriginal governance processes and study outputs will be produced with Aboriginal leadership to guide culturally appropriate policy and practice for improving health, education and social outcomes.

**Strengths and limitations of this study**

- The Western Australian Aboriginal Child Health Survey (WAACHS) linked data study enables the examination of intergenerational health and well-being pathways across multiple human service domains with up to 40 years of linked administrative life course data.
- The original WAACHS includes a large population-representative sample (1-in-6 Aboriginal children living in Western Australia in 2000); linkage between government administrative data and comprehensive interview data from the WAACHS sample will ensure exceptional granularity of information to inform analysis and interpretation of results.
- This study is driven by our Aboriginal and scientific governance processes: Aboriginal community legitimacy and guidance have been sought throughout study development and Aboriginal-led solutions and translation pathways will be enabled through sustainable community partnerships; quantitative outputs will be reviewed by Topic Expert Groups and focus groups/interviews undertaken with Aboriginal families and community members to contextualise and interpret key research findings before publication.

**STRENGTH AND LIMITATIONS OF THIS STUDY**

- Despite strong coverage from administrative data collections linked within this study, these datasets represent only a selection of available administrative data within the Western Australian and Commonwealth data ecosystems across the study period; additional linkages may be sought in the future, subject to relevant approvals.
- Although the survey data are historical, linked administrative outcome data allows us to evaluate the impact of relevant health and social policy changes up to 2020 and before the onset of the COVID-19 pandemic.

## Introduction

 Aboriginal and Torres Strait Islander Peoples (hereafter referred to as Aboriginal, as per the views of our Aboriginal advisory group in Western Australia) have maintained a successful and sustainable culture living in the harsh environments of Australia for tens of thousands of years. Since colonisation, this thriving culture has been subject to systemic disenfranchisement. Today, Aboriginal Australians face the highest levels of socioeconomic disadvantage and health inequality of any population group in Australia^1^ and lag behind other Indigenous peoples globally on many measures, including life expectancy, education and income.^2^ Despite the widely documented evidence of adversities and inequalities, the Aboriginal population is characterised by heterogeneity of experiences and cultural diversity, strength and resilience.

Addressing differential outcomes between Aboriginal and non-Aboriginal Australians is a stated, major focus of Australian governments as outlined in the 2008 Closing the Gap policy but to-date progress in closing these gaps on many stated targets has been elusive. Of the six original 2008 Commonwealth targets, early childhood education and high school completion (or equivalent attainment) rates are ‘on track’, with 86% of Aboriginal 4-year-olds enrolled in early childhood education in 2018, and 66% of Aboriginal 20–24 year-olds having attained Year 12 education or equivalent in 2018–2019. There has also been some narrowing of the gap in reading and numeracy.^3^ The remaining three targets relating to life expectancy, childhood mortality and employment outcomes are currently not on track, and urgent progress is required. This serves to illustrate both the extent of miscalculated effort in driving the level of change required for an Aboriginal population to experience equivalent health to non-Aboriginal populations in a post-colonial landscape, and the significant time lag between implementation of policy initiatives and accurate measurement of effect, estimated to be approximately 10 years.^4^ Naturally, positive change requires that the right interventions have been developed and implemented to an appropriate scale.

Any action taken to try to address the health and social disparities between Aboriginal and non-Aboriginal peoples, must consider the sociocultural, political and historical contexts in which they occur. The socioeconomic and health disparities between Aboriginal and non-Aboriginal Australians are strongly linked to the history of colonisation, discrimination and racism and the dismantling of Aboriginal families and kinship connections. The loss of culture, language and country caused deep harm and trauma, the effects of which can be seen across multiple generations. Exacerbated by ongoing systemic racism, colonisation and discriminatory public policy have had, and continue to have, a profound negative impact on social domains critical to human development, health and well-being.^5 6^

Solutions to Closing the Gap and protecting and promoting Aboriginal health and well-being are likely to be found in more progressive policy responses which value cultural identity, Aboriginal self-determination, kinship and community, and connection to Country. Understanding which investments can best support Aboriginal young people to fulfil their potential is key, and this includes resourcing areas where Aboriginal people may be doing better than other population groups.^7^ Indigenous knowledge is key in successfully developing innovative policies and programmes that respond to or prevent health and social disparities.^8^,^12^ In July 2020, the Australian Government renewed its commitment to closing human development gaps between Aboriginal and non-Aboriginal populations in partnership with The Coalition of Aboriginal and Torres Strait Islander Peak Organisations (Closing the Gap Refresh).^13^ This renewal was distinguished by a coordinated, strength-based approach to understand the antecedents (and causes) of these disparities and their longitudinal trajectory within and across generations.

To inform evidence-based policymaking in a timely manner requires not only an intricate knowledge of institutions and causal chains but also the availability of high-quality and appropriate longitudinal data to track the outcomes of populations of interest. The Western Australian Aboriginal Child Health Survey (WAACHS), a cross-sectional study conducted between 2000 and 2002, has such potential. The study provided ground-breaking evidence into the mental and physical health, educational experiences and family and community circumstances of a large, representative random sample of Aboriginal children and adolescents, aged 0–17 years.^14^,^17^ It was the first study to create an evidence base of multigenerational household data to determine the prevalence of maternal smoking, child mental health and school absence in this population, and assess the impacts of life stress and forced removal (known as The Stolen Generations policy) on Aboriginal child development, among many other firsts. Despite the volume of accumulated knowledge from the WAACHS 2000–2002 and other Aboriginal cohort studies,^18^,^24^ longitudinal data describing combined service pathways remain limited.

In this study, we have established a longitudinal cohort by linking the WAACHS with administrative data spanning a great variety of outcomes over the life course. This linkage allows us to describe the broader determinants of disadvantage over the life course, identify causal pathways for informing effective intervention and prevention across clinical and public health spheres and enable a multidisciplinary study of major health issues, and challenges to well-being and human development in the Aboriginal population. Two major issues impacting the health of Aboriginal people and the social health of entire communities is their over-representation in child protection and justice systems, a key focus of renewed Closing the Gap targets.^25^,^28^ Many studies have demonstrated the poorer health and social circumstances of child protection and justice involved children and youth in Australia.^29^,^33^

The following major health priority (HP) areas were identified through our consultation processes with Aboriginal community members and informed by health areas presenting the greatest disease burden for the Aboriginal population at different life stages.

- HP1: Mental health and trauma (including intergenerational and current trauma; mental disorders identified in health service contacts).
- HP2: Self-harm and suicidal behaviours (including self-reported and health service contacts)
- HP3: Substance use (including self-reported or carer-reported smoking and hazardous alcohol consumption, alcohol and substance use disorders identified in health service contacts).
- HP4: Infectious diseases (including skin, ears, respiratory, rheumatic heart disease and sexually transmitted diseases).
- HP5: Other non-communicable diseases (including diabetes, cardiovascular disease and chronic obstructive pulmonary disease).

These health priority areas will be viewed through a social determinants (SDs) lens as significant issues impacting the social and emotional well-being of Aboriginal children and families.

Our study is set within the wider theoretical framework of Amartya Sen’s Human Development Model.^34^ This model focuses on the development of human lives and expanding the capabilities of individuals, communities and populations to choose lives that they themselves value. It permits a focus on developmental strengths as well as an understanding of the prompts, facilitators and constraints impacting individual rights, freedom of choice and optimal outcomes. This model provides both national and global relevance to the holistic Aboriginal approach used in this study with social and emotional well-being (SEWB) as the foundation of physical and mental health.^35^

**Figure 1:**
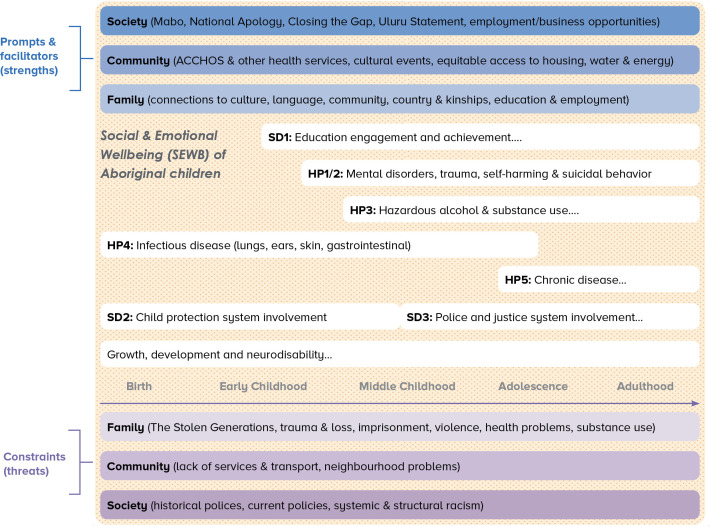
Conceptual framework for the study. HP, health priority; SD, social determinant; ACCHOS, Aboriginal Community Controlled Health Organisations

Figure 1 depicts the Human Development Model and SEWB framework as applied to this study of Aboriginal children through their development from birth into adulthood. Health priority areas (HP1–5) and social determinants (SD1–3) are shown in figure 1 as aspects that can be specifically measured by the administrative data (such as hospitalisation, child protection and justice contacts) whereas the WAACHS provides detailed developmental, family and community data collected at the time of the survey—factors that contribute to or place constraints on well-being and leading a purposeful life.

### Study aims and hypotheses

The study aims, priorities and broad research questions have been determined by our Aboriginal Steering Committee, Aboriginal stakeholder groups and supporting government agencies.

The broad aims of the study are to:

1. Understand the contribution of individual, family (intergenerational) and community-level influences on Aboriginal children’s pathways towards and away from good health and well-being, educational attainment and contact with child protection and justice involvement across the life course.
2. Identify key developmental transitions or turning points (critical events), their onset and timing, for Aboriginal children where intervention across health and social services may be most effective in improving outcomes and reducing disparities.
3. Compare service pathways for Aboriginal and non-Aboriginal children via study control groups to identify the presence of, and outcomes associated with differential treatment in otherwise similar circumstances.

The central study hypothesis is that modifiable events and situations occurring early in life (eg, housing problems, chronic infections), in the family (eg, violence, incarceration, impacts of intergenerational trauma) and across a range of health and social services (eg, systemic racism) at levels greater than those seen in the non-Aboriginal population, contribute to a disproportionately higher burden of health and social problems for Aboriginal Australians.

### Cohort description

#### Patient and public involvement (Aboriginal governance and community stakeholders)

Since WAACHS planning began in 1997, each phase has been co-designed to meet the needs of Aboriginal people and communities in Western Australia (WA) and ensure that Indigenous Data Sovereignty principles are met.^36^ For over 20 years, Aboriginal community and government representatives, and Aboriginal peak bodies, have contributed to the WAACHS and provided their support for data linkage via formal engagement channels with the research team. The authority and knowledge of longstanding partners and regional relationships will continue to be sought throughout the WAACHS Linked Data Study via our formal governance structures including an Aboriginal Steering Committee and Topic Expert Groups. As a household survey, patients were not directly involved in the study.

### Study design and participants

**Figure 2:**
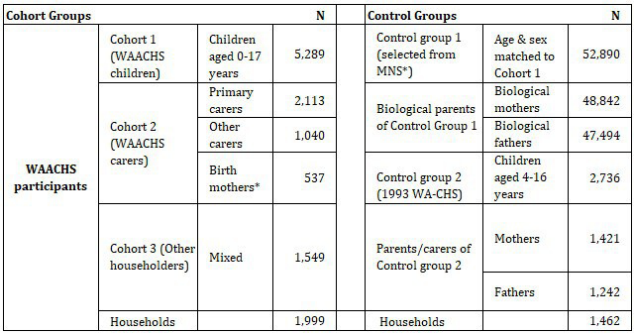
Overview of study groups. WAACHS, Western Australian Aboriginal Child Health Survey. MNS, Midwives Notification System;WA-CHS,Western Australian Child Health Survey. *Birth mothers who were not the primary carer of the child at the time of the survey

The study population for the WAACHS linked data study is represented by a set of Aboriginal child, carer and other householder cohorts sampled in the original WAACHS (2000–2002) together with two non-Aboriginal control groups comprised of children or adolescents, and their parents or carers (figure 2). The linkage of original survey data together with government administrative data has effectively created an analytical dataset spanning more than 40 years.

#### WAACHS cohorts

The WAACHS (2000–2002) was a cross-sectional random sample of one in six Aboriginal children aged 0–17 years (N=5289) living in 1999 families across WA. The Australian Bureau of Statistics estimated the resident population of Aboriginal children under the age of 18 in WA on 30 June 2001 to be 29 817, comprising 6.1% of all children in the State at that time.^14^ Sampled households were in-scope for inclusion if a parent/carer identified that one or more Aboriginal children aged under 18 years usually lived at the address. All Aboriginal children living in in-scope households were eligible to participate, along with their parents/carers. Out of a random sample of 2386 families, 1999 families (84%) consented to participate.^14^

Extensive face-to-face structured interviews with multiple informants were undertaken. In addition to interview data on the study children, primary and secondary carers provided supporting information about themselves and their communities, as well as demographic data on other householders. Data were also collected directly from the 12–17 year-olds in these households (N=1073), and from school teachers of the cohort children (N=2379). Extensive information was collected across multiple developmental domains (table 1).

**Table 1:** Research domains in the WAACHS (2000–2002) by household questionnaire

Cohort 1	Cohort 2	Cohort 1
**Child health (0–3 and 4–17 years**)	**Carers 1 (primary) and 2 (other**)	**Adolescent 12–17years**
Pregnancy and early childhood.	Language.	Aboriginal languages spoken.
General health and development.Accidents and injuries.	Participation in Aboriginal culture.Education.	Knowledge of culture and heritage.Experience of racism.
Breathing and asthma.	Employment.	Smoking.
Dental health.	Specific benefits received.	Hazardous alcohol/substance use.
Immunisation.	Perceived financial strain.	AIDS/HIV, sexual behaviour.
Other serious health problems.	Health.	Diet and nutrition.
Use of hospital/health services.	Hazardous alcohol use/tobacco use.	Asthma and hay fever.
Day care and learning.	Forced separation of children from natural family.	Emotional and behavioural problems.
Behaviour and emotional problems.Parental engagement.	Housing and accommodation.	Perceptions of family functioning.Self-esteem and self-efficacy.
Parental discipline.	Aboriginal housing standards.	Family violence.
**4–17 only**	**Carer 1 only**	Physical fighting.
Disabilities.Sleeping.	Satisfaction with neighbourhood and community amenities.	Contact with police.Experience of school.
Overnight stays from home.School and education.	Perception of community problems.Partner/spouse relationship.	School bullying.Exercise and sport.
Social and recreational activities.Mental health and well-being.	Social and religious supports.Family life stress events.	Sense of safety.Religion and spiritual beliefs.
Specific symptoms and risk behaviours.	Positive family interaction (resiliency factors).	Peer activities.Availability of a confidante.
Burden and severity of mental health.	Community strengths.	Hopes for the future.

WAACHSWestern Australian Aboriginal Child Health Survey

There are three cohorts within the WAACHS linked data study representing 10 489 unique individuals with any linked data (noting some cross-membership across Cohorts 1, 2 and 3). While Cohort 1 (the WAACHS children) is the primary focus, Cohort 2 (all WAACHS carers) is critical for understanding the developmental circumstances of the children (data on children and carers are linked). The Aboriginal carers in Cohort 2 are also important for examining health outcomes in adulthood, where follow-up extends into middle age. Cohort 3 (all other members of the household) will serve to provide further contextual information.

Cohort 1 (WAACHS children) comprises 5289 children aged 0–17 years, on whom data were directly gathered at the time of the survey (born 1982–2001), and who were aged 18–37 years by December 2019. Only one child was not linked to any administrative dataset.

Cohort 2 comprises 2113 primary carers and 1040 other carers of study children (including grandparents) and 537 additional biological mothers. Of the 5289 study children, 1002 had primary carers who were not their biological mothers. For these children, the interview team asked the primary carers to volunteer the names of the biological mothers. The missing responses and the removal of duplicates resulted in the 537 biological mothers of study children becoming part of the linkage. In total, 3690 members of Cohort 2 were linked to at least one administrative dataset.

Cohort 3 comprises 1549 other household members in the WAACHS including carers who did not complete their survey forms, non-Aboriginal children, other relatives and non-relatives. All of these cohort members were linked to administrative datasets but had no survey data.

#### Control groups

There are two control groups:

Control group 1 (non-Aboriginal) (N=52 890) is a random sample of non-Aboriginal children from the statutory WA Midwives Notification System matched to Cohort 1 on gender and age at a ratio of 10:1–based on rate ratios in offending and child protection^27^ outcomes between Aboriginal and non-Aboriginal youth in WA. Control group 1 allows comparisons of administrative outcomes for Aboriginal and non-Aboriginal children across health, education, justice and child protection domains. Selecting the control group at a 10:1 ratio allows sufficient power to describe pathways to health and developmental outcomes in both WAACHS and control children and will help inform developmental targets in the scope of the Australian Closing the Gap policy. Biological mothers (N=48 842) and fathers (N=47 494) of Control Group 1 have also been selected using genealogical data from the WA Family Connections System. Non-Aboriginal status is based on the standard-derived Aboriginal identifier used by the WA Department of Health’s Data Linkage Branch.^37^

Control group 2 (WA population) were participants in the 1993 WA Child Health Survey (WA-CHS) including 2736 4–16 year-old children (born 1976–1990) and their carers in 1462 households.^38^ The WA-CHS was a precursor of the WAACHS. Despite being an older cohort, a major strength of this control group is that the survey data contains many of the same measures of family environment and psychosocial functioning used in the WAACHS, providing the capacity to compare health trajectories for similar circumstances as captured by these measures (eg, life stress events). Note that this control group excludes Aboriginal children living in country areas for logistical reasons but does include a small number of Aboriginal children living in the Perth metropolitan area with insufficient statistical power to enable reporting.

### Linked state government administrative data

Administrative data have been linked and extracted for all study groups by the WA Department of Health Data Linkage Branch. The data linkage methodology in WA is rigorous and consistent with international standards for matching, quality control and confidentiality (separation) principles.^39^ Data are linked using probabilistic matching to create a dynamic master linkage key (unique identifier) within the system.^38^ Survey data is matched with administrative data for individuals using a unique study identifier. The approved data collections are:

- WA Department of Health: Hospital Morbidity Data Collection (1970–2019), Mental Health Information System – Ambulatory Data (1966 –2019), Emergency Department Data Collection (2002–2019), Midwives Notification System (1980–2019); Cancer Registry (1982–2019; carer groups only) and Drug and Alcohol Office (1983–2005; carer groups only).
- Datasets held at the WA Data Linkage Branch: Birth Registrations (1945–2019), Death Registrations (1969–2019) and the Australian Bureau of Statistics (ABS) Cause of Death Unit Record File (1969–2017), WA Register of Developmental Abnormalities – Birth Defects and Cerebral Palsy Registers, and Intellectual Disability: Exploring Answers Database (each 1980–2019), and the Electoral Roll (1988–2019).
- WA Department of Education (public schools data): School attendance, authorised and unauthorised absences, and suspension, National Assessment Program Numeracy and Literacy Program (NAPLAN) (2008–2014).
- WA Department of Communities. Child protection files (notifications, investigations, substantiations) and out-of-home care (1994–2015 and records up to 2019 for those already in the system by 2015).
- WA Department of Justice. Courts and sentencing (2000–2015), custodial and community corrections - adult (1985–2014) and juvenile (1995–2014) along with records to 2019/2021 for those already in the system by 2015 for courts and 2014 for corrections (Note that at the time of manuscript submission, the Team had requested that the Department of Justice review the juvenile community corrections dataset and thus linkage rates are not reflected in table 2).
- WA Police (links from a smaller, pre-existing cohort—young people aged 10–25 years from 1990 to 2015 contact with police for any crime incident that involved alcohol). (An incident may be flagged as alcohol-related in the Incident Management System if the attending officer is of the opinion that alcohol was related in any way to the incident. These include but are not limited to the offender or victim’s level(s) of intoxication at the time of the incident and may include circumstances such as identifying open or used alcohol receptacles, smelling alcohol at the scene, observing an individual affected by alcohol or receiving alcohol-related information from victims, offenders or a third party, such as witnesses. The absence of any of these circumstances does not necessarily preclude that alcohol was a contributing factor to the incident).

Linkage numbers and rates are shown in table 2 (The linkage rates in Table 2 are based on data supplied with some preliminary cleaning applied but these linkage numbers and rates could change in future analysis if invalid records are identified, or exclusions are made. Where data was supplied for 2021-2022, the linkage rates have been adjusted back to December 2019). These rates reflect the proportion of individuals that have a record in each of the administrative data collections. It is important to recognise that not all the data collections cover the full life course of each individual and therefore the proportions do not necessarily reflect population representative rates (‘incomplete coverage’). Linkages to some data collections were not requested due to being out of scope (eg, Education data for Cohorts 2 and 3) or low relevance due to age (eg, Cancer Registry data for Cohort 1). Linkage rates are also impacted by other factors such as interstate migration or attendance at non-government schools. Online supplemental figure 1 depicts the administrative datasets along a timeline, that also shows survey dates. Online supplemental figure 2 illustrates the restricted coverage in the non-health datasets for Cohort 1 by their year of birth and age at the time of the survey.

**Table 2:** Data linkage rates for WAACHS cohorts (WAACHS children, carers and other householders) for the study period to December 2019

WA government administrative data collection	Year range covered by data collection	Cohort 1(children)N=5289*	Cohort 2 (parents/carers)N=3690*	Cohort 3 (other householders)N=1549*
		N (%) with linked records	N (%) with linked records	N (%) with linked records
Death registrations	1969–2019	104 (2.0)	481 (13.0)	256 (16.5)
ABS cause of death	1969–2017	89 (1.7)	396 (10.7)	236 (15.2)
HMDC (hospitalisations)	1970–2019	4947 (93.5)	3513 (95.2)	1394 (90.0)
MHIS (mental health ambulatory)	1966–2019	1909 (36.1)	1185 (32.1)	446 (28.8)
EDDC (emergency presentations)	2002–2019	5025 (95.0)	3338 (90.5)	1343 (86.7)
MNS - midwives—own birth	1980–2019	4846 (91.6)	177 (4.8)	510 (32.9)
MNS - midwives—as a mother†	1980–2019	1467 (27.7)	2259 (61.2)	not linked
Birth registrations—own birth	1945–2019	4836 (91.4)	2682 (72.7)	not linked
Births—as a father†	1945–2019	1103 (20.9)	887 (24.0)	not linked
Births—as a mother†	1945–2019	1436 (27.2)	2384 (64.6)	not linked
WARDA (birth defects)—own birth	1980–2019	207 (3.9)	-‡	24 (1.5)
WARDA (birth defects)—as a mother†	1980–2019	101 (1.9)	373 (10.1)	not linked
WARDA (cerebral palsy)—own birth†	1980–2019	24 (0.5)	0 (0.0)	-‡
WARDA (cerebral palsy)—as a mother	1980–2019	10 (0.2)	35 (0.9)	not linked
Drug and Alcohol Office	1985–2005	not linked	121 (3.3)	45 (2.9)
WA Cancer Registry	1982–2019	not linked	342 (9.3)	100 (6.5)
IDEA—intellectual disability††	1980–2019	269 (5.1)	-‡	17 (1.1)
WA Electoral Roll	1988–2019	3227 (61.0)	3302 (89.5)	1185 (76.5)
WA Department of Education (public schools)				
Attendance	2008–2014	2675 (50.6)	not linked	not linked
Suspension	2008–2014	1064 (20.1)	not linked	not linked
NAPLAN	2008–2014	1680 (31.8)	not linked	not linked
WA Police (contacts as victim, offender or both in alcohol-related crime incidents)	See below§	1389 (26.3)	64 (1.7)	160 (10.3)
Department of Communities (Child Protection)¶				
Maltreatment notifications	1994–2015/2019	1840 (34.8)	53 (1.4)	170 (11.0)
Periods of (out-of-home) care	1994–2015/2019	422 (8.0)	12 (0.3)	43 (2.8)
Protection orders	1994–2015/2019	208 (3.9)	-‡	19 (1.2)
Department of Justice (Courts and Corrections)**				
Court—any criminal charge	2000–2015/2019	2847 (53.8)	2102 (57.0)	856 (55.3)
Juvenile corrections—custodial	1995–2014/2019	549 (10.4)	21 (0.6)	85 (5.5)
Adult corrections—community	1985–2014/2019	1060 (20.0)	1473 (39.9)	602 (38.9)
Adult corrections—custodial	1985–2014/2019	843 (15.9)	827 (22.4)	432 (27.9)

* Totals allow for a small amount of cross-membership across Cohorts 1,2 &3

† Records of a cohort member’s child with a birth defect or cerebral palsy

‡ Cells with values > 0 and ≤10 have been suppressed to preserve confidentiality

§ Records linked for the alcohol-related harm project (police records from 1990-2015 where victim or offender is aged 10-25 years and an alcohol flag is present; data from the police incident management system)

¶ Child protection records are only included up to 2019 for those already in the system by 2015.

** Dept. of Justice records are only available up to 2019 for those individuals who first offended before 2016 for courts and before 2015 for corrections data.

†† Includes some cases of children with autism, borderline ID and with insufficient information;

ABSAustralian Bureau of StatisticsEDDCEmergency Department Data CollectionHMDCHospital Morbidity Data CollectionIDEAIntellectual Disability Exploring AnswerMHISMental Health Information SystemMNSMidwives Notification SystemNAPLANNational Assessment Program Numeracy and Literacy ProgramWAWestern AustraliaWAACHSWestern Australian Aboriginal Child Health SurveyWARDAWA Register of Developmental Abnormalities

### Key indicators

Indicators will include the timing (age), frequency and length of stay for hospitalisations, emergency department presentations and mental health ambulatory programme admissions, diagnoses and comorbidity, along with patterns of service use over time and across generations. In addition, multiple measures of health status, health behaviours and health service use obtained from the WAACHS and WA-CHS survey data will be examined. Social determinant measures from linked administrative datasets include:

SD1: school education. Educational attainment variables will include school attendance rates, authorised and unauthorised absences, meeting NAPLAN National Minimum Standards^40^ and raw scores converted to equivalent year levels. Suspension records are also available.

SD2: child protection involvement. Key indicators for child protection include notifications, substantiated and non-substantiated maltreatment, type of maltreatment and time spent in out-of-home care and/or subject to a care or protection order.

SD3: justice involvement. Life-course dimensions of offending include the prevalence and frequency of offending, persistence and escalation. Detailed data has been extracted at a charge level to provide an in-depth understanding of patterns of individual offending over the life course. This includes an examination of recidivism levels, escalation in offending (by type or intensity of offending), offence specialisation (eg, in violent offending), and offending within families and across generations.

Other social and cultural determinant variables from survey data: Risk and protective factors will be identified at the child, parent, family and community levels. Measures of risk include individual (eg, exposure to family violence or racism; intergenerational trauma), domain-specific (eg, socioeconomic vulnerability), cumulative scales (eg, number of stressors) and subgroup typologies identified by exploratory analytical approaches such as latent class and latent growth analysis which identify clusters of individual, family and community risk. Proposed protective factors include cultural adherence, family support and access to community services and housing.

### Analytical methods

Study aims will be achieved through a range of analytical techniques for longitudinal data informed by developmental and biomedical science, epidemiology, criminology, economics and psychology. Statistical methods will span both standard descriptive epidemiology as well as advanced modelling techniques for examining causality in longitudinal data, such as taking advantage of natural experiments (eg, policy changes) occurring across the time span and applying fixed effects models. Comparisons between Aboriginal and non-Aboriginal children will be made using relative risk ratios with advanced modelling techniques to quantify and understand health disparities.

In addition, direct government expenditure on major health issues will be calculated and compared within Aboriginal risk and resilient subgroups (defined by factors indicating vulnerability or resilience at the time of the survey) and against control groups. Non-health costs including loss of human capital due to school drop-out or missed learning—due to absences, detention and out-of-home care will also be calculated. Where possible, we will estimate cost-savings and health benefits associated if early intervention had been in place for those identified with high burden trajectories and the cost-effectiveness of re-allocating the same resources or providing optimal care across health and other services.

#### Statistical power

The WAACHS linked data study has ample statistical power to allow for complex statistical modelling. For example, the WAACHS (2000–2002) identified that approximately 24% of the cohort had a high risk of clinically significant mental health problems compared with 15% (based on data from a contemporaneous random sample of non-Aboriginal children drawn specifically for this comparison).^15^ With a sample size of 5289 WAACHS children and a 10:1 matched control group, the study would have 99% power at a two-sided p value=0.01 to detect this difference of 9 percentage points (relative risk of 1.6). If we assume these prevalence rates apply within subgroups, then we will have >80% power to detect this difference for WAACHS subgroups as small as 400. All modelling will take account of multilevel clustering in the WAACHS sample.

### Data management

All datasets are stored on the secure network drive at the The Kids Research Institute Australia, Perth, with access only granted to approved users of the data. Analysts work on-site or connect remotely to the server with VPN. A Data Manager has oversight of all datasets, cleaning and processing with each analyst working in a personal subfolder on their specific analytical tasks.

### Community voice

Interviews and focus groups using Indigenous data collection methods such as social and research-topic yarning^41^ will be undertaken with Aboriginal community members to culturally contextualise and interpret research findings. Techniques including thematic and narrative analysis may be used to identify key themes and concepts, and to interrogate the meaning of quantitative outputs within the storied accounts of Aboriginal people.

### Findings to date

#### Cohort 1 (WAACHS children)

Maternal status and perinatal health: Overall, 4846 WAACHS children (91.6%) had a Midwives record (table 3). Of these children, the mean maternal age at birth was 23.3 years, and 64.6% of mothers were in a married or defacto relationship. The mean birth weight was 3172 g. Around 1 in 10 WAACHS children with a Midwives record were born prematurely (<37 weeks) or at a low birth weight (<2500 g). More than one in five newborns (22.4%) were considered to have suboptimal fetal growth. (Note that these figures differ slightly from the proportions recorded in the original linkage with midwives’ data in 2002 because they are unweighted, and the original linkage was only possible for 87% of children due to the requirement of carer consent at the time.)^14^

**Table 3:** Selected health characteristics of the WAACHS study children (Cohort 1, N=5289)

	n	%
**Midwives’ records (N=4846; 91.6%**)		
Maternal age of birth mother		
≤19 years	1340	27.7
20–34 years	3324	68.6
≥35 years	182	3.8
Child perinatal health		
Low birthweight <2500 g	556	11.5
Premature <37 weeks gestation*	500	11.6
Apgar score at 5 min of concern (<7)*	116	2.4
Time to spontaneous breathing ≥2 minutes*	664	14.1
Proportion with optimal birth weight <85%*	1079	22.4
**Child disability (N=5289**)		
Intellectual disability	269	5.1
Birth defects	207	3.9
Cerebral palsy	24	0.5
**Hospital admissions, any (N=5289**)		
Birth to age 1	2896	54.8
Birth to age 5	3968	75.0
Birth to age 18	4664	88.2
Any non-pregnancy-related admission (all ages)	4865	92.0
**Main principal diagnosis (ICD-10 chapter)^2^ any to age 18**		
Respiratory diseases	2508	47.4
Injury and poisoning and certain other consequences of external causes	1945	36.8
Certain infectious and parasitic diseases	1694	32.0
Digestive diseases	1303	24.6
Symptoms signs and abnormal findings	1218	23.0
Conditions in perinatal period	1112	21.0
Diseases of the skin and subcutaneous tissue	1072	20.3
Diseases of the ear and mastoid process	894	16.9
Pregnancy and childbirth (females only, n=2609)	534	20.5
**Mental health outpatient services (N=5289**)		
Any mental health outpatient service record (including referral)	1909	36.1
Adult services	1640	31.0
Child and Adolescent Mental Health Services	702	13.3
Admission for treatment in ambulatory programme	685	13.0
Adult services	433	8.2
Child and Adolescent Mental Health Services	362	6.8
**Deaths (n=104; 2.0%**)		
Age at death		
0–10 years	12	11.5
11–20 years	40	38.5
21-30 years	40	38.5
31–40 years	12	11.5

* Proportions exclude cases with missing values.

ICDInternational Classification of DiseasesWAACHSWestern Australian Aboriginal Child Health Survey

Child disability: 5% of Cohort 1 members had a record of an intellectual disability (ascertained via the Disability Services Commission or services providing support to children with disabilities in WA schools). Previous research in WA has found that children of Aboriginal mothers are twice as likely to have an intellectual disability than children of mothers of non-Aboriginal ancestry.^42^ Less than 4% of the cohort had a birth defect, and 0.5% had cerebral palsy recorded.

Hospital admissions: The child’s own birth record in the hospital data is not included if they were classified as a healthy newborn. Most of the WAACHS children (92%) had at least one non-pregnancy-related hospital admission with no difference in hospitalisation rate between genders. Over half were admitted as an infant (ages birth up to age 1), (54.8%). By adulthood, nearly half (47.4%) had been admitted at least once with a principal diagnosis (The International Statistical Classification of Diseases and Related Health Problems - 10th Revision - Australian Modification, ICD-10-AM Chapter) of respiratory disease, and more than one-third had an injury or poisoning-related hospitalisation (36.8%). Other common principal diagnosis chapters are shown in table 3. Australian studies have shown substantial inequalities in potentially avoidable hospitalisations between Aboriginal and non-Aboriginal children, most commonly respiratory and other infectious conditions and especially in the first 2 years of life.^43 44^

Emergency department presentations: Despite the restricted coverage, 95% had a least one emergency department presentation during the observation period (from 2002 to December 2019) with no gender difference observed. Each person had a 17-year window of observation but at different ages depending on birth year with a mean of 20 (median of 14, range 1–270) presentations across the total cohort.

Mental health outpatient services: Overall, 36.1% had any outpatient service record, including referral only that is, those who were referred but had no contact with services. There was no gender difference observed. Close to one in seven (13.3%) had at least one contact with (or referral to) the Child and Adolescent Mental Health Service (CAMHS) and 6.8% were admitted into at least one CAMHS ambulatory programme for treatment. Corresponding figures for adult mental health services are shown in table 3. Of those with any discharge diagnosis (or admission diagnosis if missing, n=654), the most common diagnostic category was ‘neurotic, stress-related, and somatoform disorders’ (22.4%).

Mortality register: By November 2019, 104 WAACHS study children had their deaths recorded in WA (65 males and 39 females) with just under one-third of these death registrations occurring before the age of 18. Among the 89 with an ABS cause of death code, leading causes of death were transport accidents (27%) and intentional self-harm (27%), consistent with population data for Aboriginal youth.^45^

Child protection contacts: Based on the subsample of the 5289 WAACHS study children who had full (or almost full) childhood coverage (n=2257), 44.7% had any notification record and 18.9% had any maltreatment substantiation (table 4). Of those with substantiated maltreatment, just over 40% had occurred before the age of 5; the most common type was neglect. About 10% had time in out-of-home care. These figures are consistent with prior estimates of lifetime substantiated maltreatment among Aboriginal children at 15–25% and 12% for being in care.^46 47^

**Table 4:** Selected characteristics from non-health datasets (child protection, justice and police contacts) for the WAACHS study children (Cohort 1)—subgroup analysis

	n	%
**Child protection contacts up to age 17 (N=2257 born 1994–2001**)		
Any child concern or maltreatment notification	1009	44.7
Any subsequent investigation	737	32.7
Any maltreatment substantiation	426	18.9
Birth to age 1	46	2.0
Birth to age 5	130	5.8
Birth to age 13	311	13.8
Type of (substantiated) maltreatment (any)		
Neglect	208	9.2
Sexual abuse	126	5.6
Physical abuse	109	4.8
Emotional or psychological abuse	114	5.1
Any period in out-of-home care	220	9.7
**Juvenile justice contacts aged 10–17 years (N=2572, born 1990–1997**)		
Any court record of a criminal charge	859	33.4
Age at first court appearance		
10–13 years	224	8.7
14–15 years	300	11.7
16–17 years	335	13.0
**Any criminal conviction**	**740**	**28.8**
ANZSOC group of most serious offence at first conviction*		
Acts intended to cause injury	105	14.2
Dangerous or negligent acts endangering persons	35	4.7
Unlawful entry with intent/burglary, break and enter	115	15.5
Theft and related offences	86	11.6
Property damage and environmental pollution	60	8.1
Public order offences	65	8.8
Traffic and vehicle regulatory offences	174	23.5
Other offences	100	13.5
Sentence for most serious offence at first conviction*		
Custody	12	1.6
Non-custodial (eg, youth community based orders)	268	36.2
Fine	127	17.2
Dismissed	216	29.2
Other (eg, good behaviour bond)	117	15.8
Any sentence of juvenile detention	106	4.1
Any juvenile custodial record (includes remand/presentencing)	300	11.7
**Police record of involvement in an alcohol-related crime incident age 10–25 (N=2053, born 1982–1990**)		
Any record as a victim	611	29.8
Any record as an offender	371	18.1

* ANZSOC=Australian and New Zealand Standard Offence Classification(ABS Catalogue No. 1234.0); Most serious offence selected using the National Offence Index (ABS Catalogue No. 1234.0.55.001)

Juvenile justice contacts: One-third (33.4%) of the WAACHS study children born between 1990 and 1997 had contact with the courts system prior to turning 18 years of age. These 859 children appeared in 5143 criminal court cases and faced a total of 12 178 criminal charges. The mean age at first court appearance was 15.2 years (14.9 years for males and 15.8 years for females). Overall, 28.8% of the group received a juvenile criminal conviction (twice the proportion of juvenile males to females at 38.2% and 18.9%, respectively). The most serious offence for which individuals were first convicted were ‘traffic and vehicle regulatory offences’ (23.5%). The most common sentence given at first conviction was non-custodial, typically as a youth community-based order (36.2%). Few individuals received a custodial sentence at first conviction (n=12); however, by age 18, 106 juveniles (4.1%) had received a custodial sentence and just over 1 in 10 had spent time in a custodial setting, which included being held in remand pre-sentencing.

WA police records of crimes involving alcohol: Of the 2053 Cohort members born between 1982 and 1990, 802 (39.1%) were involved in crime incidents that were alcohol-related either as a victim of a crime or as an offender. Two-thirds of victims were females (66.1%) while three-quarters of offenders were males (75.5%). Most alcohol-related offences were assaults (67.1%).

School suspension (public schools only): Of the 3236 Cohort members with public school education records (born between 1991 and 2001), 1064 had any record of suspension—one-third of all Cohort members in the subgroup. As a proportion this represents a conservative estimate as not all students attended public schools: 85% of Aboriginal students in WA went to public schools in 2001^16^ and the number of school years covered by the data ranged from 1 to 7 depending on year of birth. 62% of the 1064 with at least one suspension record were males.

### Strengths and limitations

This study has several major design strengths that distinguish it from other Aboriginal cohort studies and data resources: (1) exceptional information granularity through linkage to the face-to-face interview data about the original random sample of 5289 Aboriginal children and their carers—including multilevel social and cultural factors unique to Aboriginal populations and comprehensive information on school and community contexts; (2) a large and population representative (1-in-6) sample with coverage across metro, rural and remote areas, sufficient statistical power to examine heterogeneity and an ethics approved waiver of consent for data linkage, thus avoiding additional respondent burden and biased attrition in follow-up; (3) non-Aboriginal control groups to allow comparison of outcomes and explanation of disparities; (4) the ability to examine health and well-being pathways over multiple generations and through multiple human service domains, (5) to do so with up to 40 years of linked administrative life course data and (6) the capability to describe circumstances and outcomes for both individuals and households, including sensitive topics not easily captured in survey data. Another major strength and critical aspect of this study is the Aboriginal leadership from researchers in the investigator team, the Aboriginal Steering Committee, stakeholders across government, non-government organisations and Aboriginal community-controlled organisations.

The nature of this resource also includes limitations. First, the WAACHS 2000–2002 was a cross-sectional study, limiting the ability to discern causal relationships in the survey data. Second, administrative data collections linked within this study represent only a selection of the available administrative data within the data ecosystem of WA and the Commonwealth across the relevant period, although additional linkages may be sought in future iterations of the current study, subject to relevant approvals. Third, data obtained through record linkage was collected primarily for administrative purposes, placing a limit on the depth and completeness of available data for analysis. Fourth, when examining differences in morbidity and disability between Aboriginal and non-Aboriginal groups, or within the Aboriginal cohorts, it will not be possible to account for clinician bias or cultural appropriateness of assessments that produce these diagnoses. Finally, while the WAACHS 2000–2002 included a representative sample of Aboriginal children and their families in WA at that time, not all individuals had complete coverage within each of the administrative datasets due to truncated periods of data collection, and more so for carers.

### Future plans

Under the NHMRC Synergy Grant 2023–2028, analytical work across the health priority areas is well underway. We anticipate that findings will be disseminated in a variety of formats including but not limited to ongoing feedback and in-person dialogue with our Aboriginal stakeholders, peer-reviewed journal articles, reports to government and policy briefs, websites, lay summaries, produced in multiple languages as advised by Aboriginal stakeholders, infographics, video summaries and community forums. To enable Aboriginal control of the data, all planned analysis, reporting and outputs will be reviewed by the Aboriginal Steering Committee to ensure that the output is meaningful and policy-relevant, culturally sensitive and protects confidentiality. Further meaning and contextual understanding will be sought through qualitative research including focus groups with communities and families.

In the future, the WAACHS linked data resource will be expanded by updating linkages to all currently approved WA government health and non-health datasets, and including key datasets not presently linked (such as public housing data). This study will pursue other highly desirable linkages with Commonwealth Medicare and Pharmaceutical Benefit Scheme data to provide a more comprehensive picture of health through primary care contacts and prescribing. Furthermore, up to the time of linkage, we estimate that 48% of WAACHS children had become parents to at least one child (>5000 births to WAACHS children by December 2019) and the study will ultimately seek ethical approval to link data for these children, thus extending the Resource into a third generation.

### Summary

The longitudinal data resource established by the WAACHS linked data study will enable a rare multidisciplinary examination of major health issues and disparities in the Aboriginal population, framed within the wider perspective of a human development model that supports genuine Aboriginal agency towards improved life circumstances.^34^ Guided by the National Health and Medical Research Council strategic framework (Road Map 3)^48^ and the renewed National Agreement on Closing the Gap (July 2020) and their focus on a genuine partnership between the Australian Government and Aboriginal people,^25^ this study will address priority research areas to deliver the best outcomes for Aboriginal people and communities in collaboration with Aboriginal peak bodies and community-controlled organisations. Evidence from this study will supplement community data contributing to the development of Aboriginal-led practice interventions, responding to local needs, supporting advocacy and addressing the misconception that one size fits all in policy and programme design for Aboriginal communities.

### Collaboration

The WAACHS linked data resource has a governance framework led by an Aboriginal Steering Committee with members drawn from Aboriginal community organisations and other stakeholder groups. Outputs will be developed in consultation with Topic Expert Groups drawn from a range of settings including government departments, Aboriginal community organisations and academia.

## supplementary material

10.1136/bmjopen-2024-087522 online supplemental file 1

10.1136/bmjopen-2024-087522 online supplemental file 2

## Data Availability

Third party restrictions to data access are in place owing to statutory, legal and ethical requirements. The data used in this paper are owned by each respective Government Department and have been supplied to the study team only for the purposes of this project. Data would only be available to other parties via an approved research collaboration with the study team and all necessary data access, ethics and governance permissions provided.
