# Supplementary figures and images for: Cohort profile: Understanding the influence of early life environments and health and social service system contacts over time and across generations through the Western Australian Aboriginal Child Health Survey (WAACHS) Linked Data Study
Source: BMJ Open. 2024 Oct 2;14(10):e087522. doi: 10.1136/bmjopen-2024-087522 (PMC11448163; doi:10.1136/bmjopen-2024-087522)

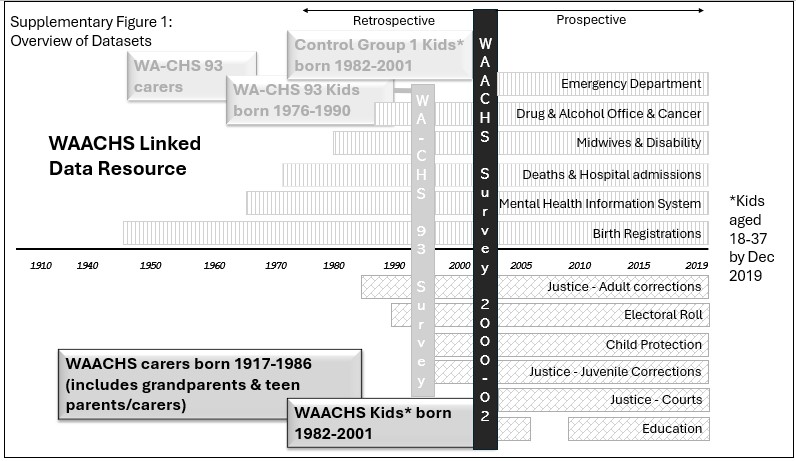

Supplement: online supplemental file 1 [file bmjopen-14-10-s001.jpg]

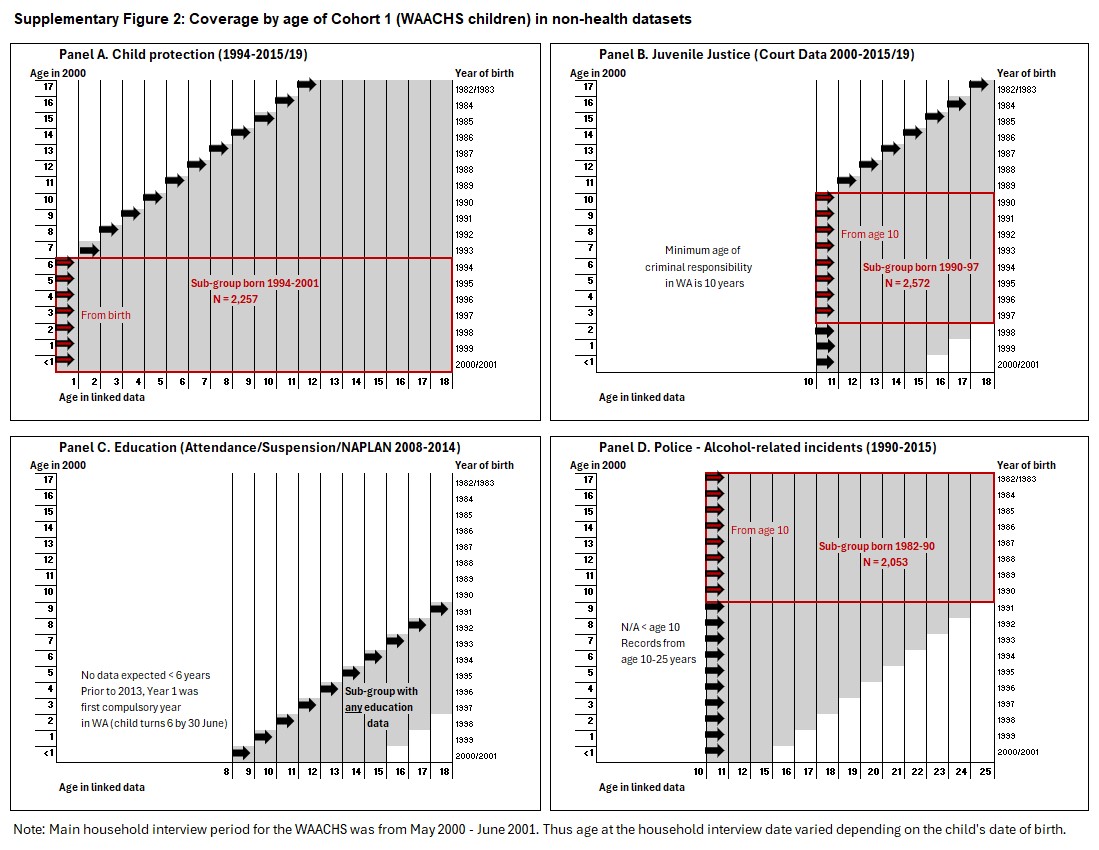

Supplement: online supplemental file 2 [file bmjopen-14-10-s002.jpg]
