# Supplementary material for: Cohort profile: Understanding the influence of early life environments and health and social service system contacts over time and across generations through the Western Australian Aboriginal Child Health Survey (WAACHS) Linked Data Study
Source: BMJ Open. 2024 Oct 2;14(10):e087522. doi: 10.1136/bmjopen-2024-087522 (PMC11448163; doi:10.1136/bmjopen-2024-087522)
Supplement: Supplementary file 3 [file bmjopen-2024-087522.reviewer_comments.pdf]

PEER REVIEW HISTORY

BMJ Open publishes all reviews undertaken for accepted manuscripts. Reviewers are asked to complete a checklist review form and are provided with free text boxes to elaborate on their assessment. These free text comments are reproduced below.

ARTICLE DETAILS

**TITLE (PROVISIONAL)**	Cohort profile: Understanding the influence of early life environments and health and social service system contacts over time and across generations through the Western Australian Aboriginal Child Health Survey (WAACHS) Linked Data Study
**AUTHORS**	Mitrou, Francis; Milroy, Helen; Coffin, Juli; Hamilton, Sharynne; Brennan-Jones, Christopher; Schurer, Stefanie; Davis, Elizabeth; Richmond, Peter; Passmore, Hayley; Pearson, Glenn; Brown, Alex; O'Donnell, Melissa; Bowen, Asha; Azzopardi, Peter; Conigrave, Katherine; Downs, Jenny; Cooper, Matthew; Ramsey, Kathryn; Ferrante, Anne; Johnson , Sarah; Cave, Leah; Vlaskovsky, Philip; Hopkins, Katrina; D'Antoine, Heather; Wilkes, Ted; Zubrick, Stephen

VERSION 1 - REVIEW

**REVIEWER NAME**	Sjöholm, Pauline
**REVIEWER AFFILIATION**	University of Turku
**REVIEWER CONFLICT OF INTEREST**	No competing interests
**DATE REVIEW RETURNED**	17-May-2024

**GENERAL COMMENTS**	Thank you very much for the opportunity to review this paper that describes a profile of the WAACHS Linked Data Study. It is an impressive study with good, representative coverage of the Aboriginal population in Western Australia and has extensive links to many administrative databases covering health and wellbeing, including hospital records, child protection services and the justice system. According to the authors, it has the potential to provide information about ‘critical events’ during the life course and thus provide better means for early interventions. Here are some comments and thoughts after reading the paper.<br>The paper is very long. A ‘cohort profile’ –paper should in my opinion provide an overview of the cohort and the purposes of the study and some general outlines of future plans. However, it is quite difficult to grasp what is actually planned and why, as there is so much material in the paper. That is why I believe that the paper needs some restructuring to make it easier to read. After careful restructuring the paper could work as a great tool for other researchers as a reference in future papers.<br>There are many aims mentioned, such as understanding the contribution of family and community-level influences on health pathways and identifying transition points as well as comparing service pathways. After reading the paper, it remains unclear how these aims will be achieved. Are you going to do trajectory analyses about service contacts? Will you be grouping the participants in other ways to be able to compare those at higher risk with those at lower risk? How will you be linking the intergenerational data?<br>Some more specific comments. You mention the Closing the Gap – report a few times in the paper. I think you could introduce the report

	earlier in the introduction to make it more coherent when you later make references to it. Some of you references seem a little old, as I believe there is newer literature available to describe the current situation. For example references 1 and 6 are more than 15 years old. You refer to many Indigenous cohorts in the introduction, maybe consider referring to the ABC from the NT as well?<br>Figure 1 is not available to see. Is this a technical issue or did you forget to insert it? The introduction is too long and I'm unsure whether the theoretical framework and the description of the socioeconomic circumstances need so much space. Surely it could be summarized quite substantially.<br>Some of the early life predictors you mention, such as intergenerational trauma, seem quite abstract and difficult to quantify. How do you measure these factors?<br>Consider restructuring table 1 as there is a lot of information listed with an unclear structure. Is it possible to make a flow chart of the sample selection and add research domains to the flow chart?<br>The role of cohort 2 needs clarification. Will you be linking the collected data of early life factors with the life course measures of cohort 1? How will this be done? What is the purpose of cohort 3? Why do you have two control groups? Especially the purpose of control group 2 remains unclear. It is confusing that some Aboriginal children were included but others not in cohort group 2.<br>The reference to data linkage principles is 25 years old. Is it still relevant? (reference 38).<br>Are the health priority areas in a specific order or all equally important?<br>What do you mean with natural experiments in this context (analytic methods)? What standard is the classification of relative isolation based on (sociodemographic characteristics of cohort 1)?<br>You give findings to date only for cohort 1. Are there no findings for the other cohort or how do you justify this? I can't understand the phrase "Almost two-thirds were admitted as an infant (ages birth up to age 1), excluding healthy newborns (64.2%)." Who are you excluding?<br>Table 3 lists selected health characteristis of the study children. One of the characteristics is "time to spontaneous breathing > 2 sec". This seems odd to me, as I believe it is normal and common for an infant to take 10-15 sec to start spontaneous breathing.<br>The chapter for findings is also too extensive and needs substantial summarizing. Some of the material could be moved to a 'supplemental materials' –section to make the entity more readable.<br><br>In conclusion, to get the most of this unique study, I would carefully revise the paper, remove all repetitive sections, summarize all sections and restructure the paper so that it would form a more coherent entity that a lot of people could use as a reference in the future, as it seems that there is potential to publish a lot of research articles within this framework.

**REVIEWER NAME**	Hanly, Mark
**REVIEWER AFFILIATION**	University of New South Wales, Centre for Big Data Research in Health
**REVIEWER CONFLICT OF INTEREST**	NA
**DATE REVIEW RETURNED**	31-May-2024

GENERAL COMMENTS	Thank you for the opportunity to review this cohort profile, which was well-written, clearly motivated, and thoroughly summarised. Congratulations on the enormous amount of work that has gone into bringing together this outstanding linked data resource and good luck for the many future applications.<br><br>The manuscript could be published as is; I have made some very minor comments below for your consideration.<br><br>Given the scale and complexity of the linked data assets perhaps add a few lines on data management plans, e.g. data processing/cleaning and data storage (use of a secure environment?).<br><br>The study design means there is follow up to different ages depending on children's age at baseline (e.g. only 1,602 had full childhood coverage for the child protection contacts analysis on p20). Is there some way to visually summarise the coverage for different age groups and key datasets in a supplementary figure/table? Perhaps showing age at baseline on the vertical access, year of age on the horizontal access and different coloured horizontal bars to represent coverage for different datasets over time.<br><br>Possible typos:<br>- Page 19, line 12: 9 percentage points rather than 9%.<br>- Page 26, line 38: waiver (on) of consent?

VERSION 1 – AUTHOR RESPONSE

**Reviewer 1 comments**	
The paper is very long. A 'cohort profile' –paper should in my opinion provide an overview of the cohort and the purposes of the study and some general outlines of future plans. However, it is quite difficult to grasp what is actually planned and why, as there is so much material in the paper. That is why I believe that the paper needs some restructuring to make it easier to read. After careful restructuring the paper could work as a great tool for other researchers as a reference in future papers.	Response: We thank the reviewer for their careful consideration of the manuscript and for pointing out areas that need further clarity and areas for reducing material. Whilst some additional sentences have been added in response to this feedback, more content has been removed overall with the aim of reducing repetition. The total word count has been reduced substantially from 7,652 words in the original submission to 6,246 words.<br><br>The paper has had some restructuring but not major. Hopefully this, together with additional edits outlined below will make the paper easier to read.<br><br>Reference to page numbers below are in the clean revised manuscript.<br><br>Changes.

	The health priority areas section has been moved into the Introduction before Figure 1 for better flow. See p.6-7.<br><br>Two paragraphs on child protection and justice have been removed and summarised at the end of para 6. See p.6<br><br>The section on the 'Sociodemographic characteristics of Cohort 1(the WAACHS children)' has now been removed, and brief reference made to important figures in the study design and participants section. See p. 9.<br><br>There is no longer a discussion section but a new section on Future plans has been created merging what was 'Outputs and Dissemination' with 'Future Directions' to make the planned activities clearer. This is now positioned after 'Strengths and limitations'. See p.22.<br><br>There is now a brief summary at the end. See p.22-3.
There are many aims mentioned, such as understanding the contribution of family and community-level influences on health pathways and identifying transition points as well as comparing service pathways. After reading the paper, it remains unclear how these aims will be achieved.<br><br>Are you going to do trajectory analyses about service contacts?<br><br>Will you be grouping the participants in other ways to be able to compare those at higher risk with those at lower risk?<br><br>How will you be linking the intergenerational data?	Response: It is true that the study aims are broad. As we are working across health priority areas and through a social determinants lens, there are many research questions that are to be refined by the Investigators and Aboriginal Steering Committee. From these specific and measurable RQs, a detailed analytic plan will be developed utilising the most appropriate methods. Therefore, yes there will be trajectory analyses and grouping participants in different ways by risk levels. Types of potential analysis has been outlined in the section on analytic methods (see p.15-16). The data from children and their parents/carers are already linked and so the infrastructure is in place.<br><br>Change. Additional information p.9 about Cohorts and the phrase "data on children and carers are linked".

Some more specific comments.<br><br>You mention the Closing the Gap – report a few times in the paper. I think you could introduce the report earlier in the introduction to make it more coherent when you later make references to it.	Response: Closing the Gap is the focus of the second paragraph in the Introduction. It couldn't be mentioned any earlier than it already is. This is a reference to the original 2008 goals. And then in paragraph 4, we introduce the July 2020 renewed commitment with the Closing the Gap Refresh. On review, we cannot see that changing the flow of the first 4 paragraphs would improve the manuscript.<br><br>Change. The first sentence in para 2, p.5 has been reworded to make it clearer that we are referring to government policy.<br><br>.
Some of you references seem a little old, as I believe there is newer literature available to describe the current situation. For example references 1 and 6 are more than 15 years old.	Response: Gracey and King (ref #1) is a seminal paper and so it is highly relevant. It has been retained. The Carson reference is also highly cited and has been retained but with reference to the latest publication date in 2020.<br><br>Change. We have updated ref #6.
You refer to many Indigenous cohorts in the introduction, maybe consider referring to the ABC from the NT as well?	Response: Yes, we are certainly familiar with the ABC study and it was an oversight.<br><br>Change. We have added a reference to the 2017 cohort profile paper for the ABC study by Sayers et al. See Ref #18.
Figure 1 is not available to see. Is this a technical issue or did you forget to insert it?	It was a requirement of submission to include Figure 1 as an additional document, which we can see as visible on the last page of the proof document. Perhaps this is why it was missed, because it did not appear in the body of the manuscript?
The introduction is too long and I'm unsure whether the theoretical framework and the description of the socioeconomic circumstances need so much space. Surely it could be summarized quite substantially.	Response: We have reduced the Introduction by removing the 2 paragraphs about child protection and justice, and including 2 sentences with essential references at the end of para 6 (Commencing "In this study…") We are satisfied that this level of detail will be more important in publications that are specifically focused on these topics. The paragraph on the theoretical framework has also been trimmed.

	Change. See 2 sentences added on p.6.
Some of the early life predictors you mention, such as intergenerational trauma, seem quite abstract and difficult to quantify. How do you measure these factors?	Response: An important observation. One key measurable aspect of intergenerational trauma will be the impact of forced separation from families among parents and grandparents (the Stolen Generations) on children in the household. The WAACHS was the first population study of Australian Indigenous families to demonstrate multigenerational impacts of forced separation on child development outcomes. Intergenerational trauma may also stem from a set of adverse childhood experiences, aside from being part of the Stolen Generations. Trauma impacts will be quantified in measures of family violence, mental health and substance use issues, welfare reliance and incarceration at the carer level, and potentially at the descendent (child-level) in this study. Our Aboriginal investigators and Steering Committee will guide us in quantifying and reporting on intergenerational trauma impacts.<br><br>No change.
Consider restructuring table 1 as there is a lot of information listed with an unclear structure.	Response: It is difficult to know how to restructure this table without specific feedback as to what is unclear. Please provide more detailed suggestions if required.<br><br>Change: The only things we could think to do is to add the total N in each of the three cohorts in the column headings, and to replace the N/As with 'not linked'.
Is it possible to make a flow chart of the sample selection and add research domains to the flow chart?	Response: As this study is based on an existing cohort, no new sample is being selected, a flow chart doesn't seem like the right visualisation for this paper. The sample selection for the original WAACHS sample is described in detail in Volume 1 of the original findings. See Zubrick et al. 2004 (ref #14). In addition, the research domains cut across the cohort and control groups, especially as family-level and intergenerational effects are being examined. However, we have provided an additional diagram that may help.

	Change. See Figure 2, referred to in page 8.
The role of cohort 2 needs clarification.	Response: Cohort 2 are the carers of Cohort 1 and for that reason, information about these carers is essential for understanding the human development 'capital' available to children in the study and the family environment. The primary carer was the main source of information about the family and community, with an extended set of questions on the Carer 1 form.<br><br>The Aboriginal carers in Cohort 2 are also a focus themselves, due to their older age and ability to track them into middle age in the administrative data, especially in respect to chronic disease outcomes (HP5).<br><br>Change. The following sentence has been added on p.9. "Whilst Cohort 1 (the WAACHS children) are the primary focus, Cohort 2 (all WAACHS carers) are critical for understanding the developmental circumstances of the children (data on children and carers are linked). The Aboriginal carers in Cohort 2 are also important for examining health outcomes in adulthood, where follow-up extends into middle age. Cohort 3 (all other members of the household) will serve to provide further contextual information."
Will you be linking the collected data of early life factors with the life course measures of cohort 1? How will this be done?	Response: The survey data from the WAACHS has already been linked with the administrative data sets. We have made this clearer.<br><br>Action: A slight modification has been made to the first paragraph on linked state government administrative data.<br><br>"Administrative data have been linked and extracted for all study groups… Survey data is matched with administrative data for individuals using a unique study identifier." See p.11.
What is the purpose of cohort 3?	Response: Cohort 3 is comprised the other householders at the time of the survey (i.e. not children, and not carers) and whilst the group is mixed in its composition, Cohort 3 may serve several purposes. These individuals complete the picture of the household at the time of the survey, and linked administrative data will show if there are other

	exposures to illness in the family, death or incarceration impacting on other family/household members. Certain individuals within Cohort 3 may also be of interest, such as older siblings (aged > 17 at the time of the survey).<br><br>Action. See response above – additional text on page 9.
Why do you have two control groups? Especially the purpose of control group 2 remains unclear.	The two control groups provide two different types of comparison. Control group 1, age and sex matched to Cohort 1 (WAACHS children) at a ratio of 10:1 provide the ability to make comparisons at the same point in time, and for outcomes that are vastly overrepresented in Aboriginal children. However, Control group 1 only has linked administrative data, whereas Control group 2 also has detailed survey data and thus can compare the effects of similar family circumstances (e.g. SES, stressors) on child outcomes for Aboriginal children versus a population sample. As an older cohort, we do not have linked child protection and justice data for control group 2 because the administrative data are not available that far back in time.<br><br>No change.
It is confusing that some Aboriginal children were included but others not in cohort group 2.	I think the reviewer means control group 2, participants in the 1993 WA Child Health Survey? Please refer to ref #38 for more information about the methodology for this survey (Appendix C). This 1993 survey was not intended to be representative of Aboriginal children in the State and thus was not designed for travel to remote areas for data collection. For logistical reasons of survey administration, Aboriginal children were included in the sample in the Perth metropolitan area in proportion to population. However, Aboriginal children were not present in the sample at high enough numbers to support population estimates. Further to this, Aboriginal leaders were consulted in the design phase for the 1993 survey and requested an Aboriginal specific survey be designed and implemented at a later date. This became the WAACHS.<br><br>Change: Page 11 – added “for logistical reasons”

The reference to data linkage principles is 25 years old. Is it still relevant? (reference 38).	Response: Yes, it is still relevant and is frequently cited in data linkage research as the foundational paper for WA and Australian data linkage systems.<br><br>No change.
Are the health priority areas in a specific order or all equally important?	Response: all health priority areas are equally important. Within each area – the Investigators and Steering Committee will prioritise specific research questions and inevitably there is overlap with clustering of circumstances and co-morbidities.<br><br>No change.
What do you mean with natural experiments in this context (analytic methods)?	Response: As the data and the lives of the Aboriginal children (and carers) in the study span decades, we aim to identify legislative/policy change in government sectors such as housing or child protection that may have impacted pathways. These events can be modelled as natural experiments using methods from the field of economics that can estimate causal impacts of these events/policies.<br><br>Change: Added e.g. policy change in brackets. See p.16.
What standard is the classification of relative isolation based on (sociodemographic characteristics of cohort 1)?	Response: A new classification of remoteness – the Level of Relative Isolation (LORI) – was designed for the WAACHS. The LORI is based on a product from the National Key Centre for Social Application of Geographic Information Systems at Adelaide University (GISCA) called ARIA++. The ARIA++ is an extension of ARIA (the Accessibility/ Remoteness Index of Australia), which has been widely adopted as the standard classification of remoteness in Australia.<br><br>Change: This section has been removed.
You give findings to date only for cohort 1. Are there no findings for	Response: Cohort 1 – the Aboriginal children in our study are the primary cohort of interest in this Study and thus we only present initial findings for this group in this paper. Presenting results for the other cohorts is not feasible in the

the other cohort or how do you justify this?	context of this manuscript, although as we publish further scientific outputs, these cohorts will be described. As the reviewer has already pointed out below, the Chapter for findings is too extensive and has asked that we summarize.<br><br>No change.
I can't understand the phrase "Almost two-thirds were admitted as an infant (ages birth up to age 1), excluding healthy newborns (64.2%)." Who are you excluding?	Response: We are excluding records of the infant's own birth if they were classified as being a healthy newborn. Since submitting the manuscript, we discovered an error that meant that not all healthy newborns had been excluded, thus the correct figure is now 54.8%.<br><br>Change: We have removed the phrase "excluding healthy newborns" and added a new sentence: "The child's own birth record is not included if they were classified as a healthy newborn". See page 17.
Table 3 lists selected health characteristics of the study children. One of the characteristics is "time to spontaneous breathing > 2 sec". This seems odd to me, as I believe it is normal and common for an infant to take 10-15 sec to start spontaneous breathing.	Response: Thank you for pointing out this error. You are right, the metric here is not seconds but >= 2 minutes.<br><br>Change: Corrected in Table 3.
The chapter for findings is also too extensive and needs substantial summarizing. Some of the material could be moved to a 'supplemental materials' –section to make the entity more readable.	Response: Information in the Findings section has been reduced – partly by summarising, and partly by not citing table figures in text. We think it is important to keep this material in the manuscript as it provides a high-level view across datasets. Please note that some of the Figures in Table 3 and 4 have been updated due to decisions around inclusion criteria.<br><br>Change. See changes in Tables and reduced text.
In conclusion, to get the most of this unique study, I would carefully revise the paper, remove all repetitive sections, summarize all sections and	Response: Hopefully the changes make for an easier read. We certainly do plan for many future publications that will refer to this paper as the foundation and so it needs to be

restructure the paper so that it would form a more coherent entity that a lot of people could use as a reference in the future, as it seems that there is potential to publish a lot of research articles within this framework.	clear. We thank the reviewer for their very helpful comments.<br><br>No change.
**Reviewer 2 comments**	
The manuscript could be published as is; I have made some very minor comments below for your consideration.<br><br>Given the scale and complexity of the linked data assets perhaps add a few lines on data management plans, e.g. data processing/cleaning and data storage (use of a secure environment?).	Response: Data management is certainly a key aspect of this project to ensure secure and safe access, and consistency across users in cleaning, processing and specific analyses.<br><br>Change. A paragraph has been added describing data management on page 16.<br><br>"All datasets are stored on the secure network drive (at the Telethon Kids Institute in Perth, with access only granted to approved users of the data. Analysts work on site or connect remotely to the server with VPN. A Data Manager has oversight of all datasets, cleaning and processing with each analyst working in a personal sub folder on their specific analytic tasks using copies of cleaned data files."
The study design means there is follow up to different ages depending on children's age at baseline (e.g. only 1,602 had full childhood coverage for the child protection contacts analysis on p20). Is there some way to visually summarise the coverage for different age groups and key datasets in a supplementary figure/table? Perhaps showing age at baseline on the vertical access, year of age on the horizontal access and different coloured horizontal bars to represent coverage for different datasets over time.	Response: Yes, this is a challenge of working with the linked data, especially the non-health datasets. For Cohort 1 (the WAACHS children), there is complete visibility in most of the health datasets apart from the ED data. We have included 2 new supplementary figures that should help. Supp Figure 1 shows all of the linked administrative datasets against a timeline. Supp Figure 2 (Panels A-D) visualises the limits of the child protection, juvenile justice, education and police data sets and how sub-groups with complete coverage have been selected for the profile paper.<br><br>Change. See Supplementary Figure 1 and Supplementary Figure 2, as referred to on page 12.
Possible typos:<br>- Page 19, line 12: 9 percentage points rather than 9%.<br>- Page 26, line 38: waiver (on) of consent?	Done! Thank you.

VERSION 2 – REVIEW

**REVIEWER NAME**	Sjöholm, Pauline
**REVIEWER AFFILIATION**	University of Turku
**REVIEWER CONFLICT OF INTEREST**	No competing interests
**DATE REVIEW RETURNED**	03-Sep-2024

**GENERAL COMMENTS**	I thank the authors for the careful revision. In my opinion, the most important problems were assessed and I can recommend the publication of the paper in its current form.

VERSION 2 – AUTHOR RESPONSE
